# Characterisation of parasympathetic ascending nerves in human colon

**DOI:** 10.3389/fnins.2022.1072002

**Published:** 2022-12-01

**Authors:** Michaela E. Johnson, Adam Humenick, Rochelle A. Peterson, Marcello Costa, David A. Wattchow, Tiong Cheng Sia, Phil G. Dinning, Simon J. H. Brookes

**Affiliations:** ^1^College of Medicine and Public Health, Flinders University, Adelaide, SA, Australia; ^2^Colorectal Surgical Unit, Division of Surgery, Flinders Medical Centre, Bedford Park, SA, Australia; ^3^Department of Gastroenterology and Surgery, Flinders Medical Centre, Bedford Park, SA, Australia

**Keywords:** parasympathetic nervous system, ascending nerves, shunt fascicles, human, colon, LARS

## Abstract

**Background:**

In the human large bowel, sacral parasympathetic nerves arise from S2 to S4, project to the pelvic plexus (“hypogastric plexus”) and have post-ganglionic axons entering the large bowel near the rectosigmoid junction. They then run long distances orally or aborally within the bowel wall forming “ascending nerves” or “shunt fascicles” running in the plane of the myenteric plexus. They form bundles of nerve fibres that can be distinguished from the myenteric plexus by their straight orientation, tendency not to merge with myenteric ganglia and greater width.

**Aim:**

To identify reliable marker(s) to distinguish these bundles of ascending nerves from other extrinsic and intrinsic nerves in human colon.

**Methods:**

Human colonic segments were obtained with informed consent, from adult patients undergoing elective surgery (*n* = 21). Multi-layer immunohistochemical labelling with neurofilament-H (NF200), myelin basic protein (MBP), von Willebrand factor (vWF), and glucose transporter 1 (GLUT1), and rapid anterograde tracing with biotinamide, were used to compare ascending nerves and lumbar colonic nerves.

**Results:**

The rectosigmoid and rectal specimens had 6–11 ascending nerves spaced around their circumference. Distal colon specimens typically had 1–3 ascending nerves, with one located near the mesenteric taenia coli. No ascending nerves were observed in ascending colon specimens. GLUT1 antisera labelled both sympathetic lumbar colonic nerves and ascending nerves in the gut wall. Lumbar colonic nerves joined the myenteric plexus and quickly lost GLUT1 labelling, whereas GLUT1 staining labelled parasympathetic ascending nerves over many centimetres.

**Conclusion:**

Ascending nerves can be distinguished in the colorectum of humans using GLUT1 labelling combined with NF200.

## Introduction

Parasympathetic nerves to the abdominal organs are divided into vagal/cranial and pelvic/sacral pathways (Langley, 1921). The proximal portion of the large bowel is innervated by vagal parasympathetic efferent input (Dapoigny et al., 1992) with the more distal regions receiving input from sacral parasympathetic pathways. The latter originate from preganglionic neurons within S2–S4 regions of the lumbosacral spinal cord which synapse onto postganglionic neurons within ganglia of the pelvic (hypogastric) plexus, from which axons project in rectal nerves into the bowel wall (Fukai and Fukuda, 1985; Baader and Herrmann, 2003). Recently, the nomenclature of the sacral parasympathetic pathways has been challenged based on the expression of transcription factors, with a suggestion that the pelvic pathway is essentially sympathetic (Espinosa-Medina et al., 2016). However, since this interpretation has been disputed because both thoracolumbar sympathetic and sacral pathways share a spinal origin (Neuhuber et al., 2017; Horn, 2018), we will continue to use the original term of “parasympathetic” to describe the sacral outflow to the gut. While the descriptions “sympathetic” and “parasympathetic” are primarily anatomical descriptions the pathways appear to perform different functions, with sympathetic drive inhibiting colonic motility, whereas parasympathetic pathways are largely excitatory (Langley and Anderson, 1895) and play an important role in defaecation (M’Fadden et al., 1935; Knowles et al., 2001; Heitmann et al., 2021).

Stach (1971) described branches of the pelvic plexus in dogs and cats which run subserosally before entering the wall of the colon near the rectosigmoid junction using silver staining, methylene blue, cholinesterase histochemistry, and osmium/zinc iodide. From the entry point, these presumed parasympathetic branches projected for long distances up to the top of the descending colon, in the same plane as the myenteric plexus (Stach, 1971). Stach referred to these as “ascending nerves” and noted that they travel in both directions in the colon from their entry points, i.e., aborally toward the anal canal and orally as far as the transverse colon. They have a distinctive straight path along the longitudinal axis of the colon and rarely pass through myenteric ganglia. Rather, they pass over or beside ganglia with which they interconnect *via* some axons.

Ascending nerves have many characteristics in common with peripheral nerves, including the presence of myelinated fibres, endoneurial collagen, and blood vessels within the nerve trunk and a perineural sheath (Stach, 1971; Christensen et al., 1984; Fukai and Fukuda, 1984; Christensen and Rick, 1987). Since Stach’s original description a few studies have focussed on further characterising ascending nerves, with a small number assessing human clinical samples (Kumar and Phillips, 1989; O’Kelly et al., 1994; Wester et al., 1999). Studies in human tissue have shown that ascending nerves are present in equal frequency in neonatal and adult colon (Kumar and Phillips, 1989), that they contain some NOS-immunoreactive axons (O’Kelly et al., 1994), and they stain more weakly for NADPH diaphorase activity (a histochemical marker of NOS) than regular internodal strands (Wester et al., 1999).

Much remains unknown about ascending nerves, in terms of the best markers to reliably identify them, which enteric cells they synapse with and what is their function. In this study we used a panel of immunohistochemical markers in adult human tissue collected from multiple regions of the colon to further characterise ascending nerves and to determine the best markers for their reliable identification. Being able to readily identify ascending nerves will be crucial for future studies to effectively explore their functional role and the clinical significance of their disruption.

## Materials and methods

### Specimens

After obtaining written informed consent for use of colonic tissue for research purposes, samples were collected from 21 patients undergoing elective colorectal surgery for large bowel cancer (*n* = 17) or diverticulitis (*n* = 4), (Southern Adelaide Clinical Human Research Ethics approval number 207.17). Samples were taken approximately 15–30 cm away from the lesioned bowel. Data recorded from patients was restricted to sex, age, reason for operation, region of colon, date of operation and surgeon. Twelve specimens were used for full circumference analysis; these included ascending/start of transverse colon (*n* = 3), descending colon (*n* = 4), sigmoid colon (*n* = 3), rectosigmoid (*n* = 1), and rectum (*n* = 1). One sample of descending colon was excluded due to excessive damage during dissection. Six specimens were used for rapid anterograde tracing of ascending nerves with biotinamide; descending colon (*n* = 2), sigmoid colon (*n* = 4). An additional three specimens were discarded because biotinamide was applied to internodal strands rather than ascending nerves (*n* = 1 descending colon and *n* = 2 sigmoid colon). Of the 17 patients included *n* = 9 were male and *n* = 8 were female, with a median age of 68 years (range 55–83).

### Tissue collection

A 2 cm ring of tissue was cut from the healthy margin of the colonic specimen and transported from the operating theatre to the laboratory, located in the same building, in room temperature oxygenated modified Krebs solution (NaCl; 118 mM, KCl; 4.8 mM, CaCl_2_; 2.5 mM, MgSO_4_; 1.2 mM, NaHCO_3_; 25 mM, NaH_2_PO_4_; 1.0 mM, glucose; 11 mM, bubbled with 95% O_2_, 5% CO_2_, pH 7.4). The intact ring of colonic tissue was opened up longitudinally between the two antimesenteric taenia. Tissue was then pinned out in a Sylgard-lined petri dish (Sylgard Elastomer 184, Dow, Midland, MI, United States), serosal side down, allowing the mucosa and submucosa to be removed by sharp dissection.

### Rapid anterograde tracing

Tissue allocated for rapid anterograde tracing underwent further dissection to remove circular muscle from the aboral end of the specimen to reveal ascending nerves in the live tissue. When identified, ascending nerves were dissected free of the underlying tissue over a length of at least 5 mm so that rapid anterograde tracing could be performed, as previously described (Chen et al., 2015). In brief, the specimen was pinned in a sterile petri dish lined with Sylgard, with the ascending nerve led into a small Perspex chamber sealed with high-vacuum silicon grease (Auburn, NSW, Australia) and a glass coverslip. The nerve was washed repeatedly with artificial intracellular medium solution [150 mM monopotassium, L-glutamic acid, 7 mM MgCl_2_, 5 mM glucose, 1 mM ethylene glycolbis-(b-aminoethyl ether) N,N,N’,N’-tetra-acetic acid (EGTA), 20 mM hydroxyeicosapentaenoic acid (HEPES) buffer, 5 mM disodium adenosine-5-triphosphate (ATP), 0.02% saponin, 1% dimethyl sulphoxide (DMSO), 100 IU/ml penicillin, and 100 μg/ml streptomycin, 20 μg/ml gentamycin] (Tassicker et al., 1999), then the small chamber was filled with paraffin oil. A small drop of 5% biotinamide [N-(2-aminoethyl) biotinamide, hydrobromide; Molecular Probes, Eugene, OR, United States] dissolved in the same artificial intracellular solution was placed on the ascending nerve in the isolated chamber. The Krebs solution in the main chamber was replaced with sterile culture medium [DME/F12 (Sigma, St. Louis, MO, United States) with 10% v/v faetal bovine serum, 100 IU/ml penicillin, 100 μg/ml streptomycin, 20 μg/ml gentamycin, 2.5 μg/ml amphotericin, 1 μM nicardipine, and 1 μM hyoscine] and placed on an orbital mixer in a humidified incubator at 37°C in 5% CO_2_ overnight. The following morning the biotinamide and paraffin oil were carefully removed and the isolation chamber with the ascending nerve was rinsed with phosphate-buffered saline (PBS) three times to ensure that all free biotinamide was removed.

### Tissue fixation

Tissue (either from biotinamide fills or from acute specimens) was re-pinned, serosa-side up in a Sylgard-lined petri dish under maximal tension, then immersion-fixed in 4% paraformaldehyde overnight (4% paraformaldehyde in 0.1 M phosphate buffer, pH 7.2 at 4°C). The next morning, specimens were unpinned and immersed in 4% paraformaldehyde for an additional 24 h at room temperature, with light agitation, to ensure complete fixation.

### Multi-layer immunohistochemistry

Full circumference specimens were cut into three sections, each containing one taenia, before further dissection to remove as much of the circular muscle layer as possible, without damaging the underlying myenteric plexus. Preparations used for biotinamide tracing also underwent further dissection to remove circular muscle. They were then incubated in Streptavidin Alexa Fluor 488 (1:400, 0.5% Triton™ X-100) for two nights. All other preparations were rinsed in PBS then immersed in 0.5% Triton X-100 in PBS for two nights at room temperature to permeabilise the tissue. Next, each preparation was incubated in primary antibodies for three nights at room temperature, washed in PBS then incubated in secondary antibodies overnight. Finally, preparations were rinsed in PBS and equilibrated with buffered glycerol diluted with PBS (50%→70%→100%, pH 8.6) before mounting on glass slides.

As previously described (Parker et al., 2022), the full circumference preparations underwent elution to remove bound primary and secondary antisera, allowing a new layer of different markers of interest to be applied. In brief, preparations were submerged in a 2-mercaptoethanol/SDS buffer (2-ME/SDS) (Gendusa et al., 2014) and incubated in a pre-heated water bath (56°C) for 1 h with agitation at 60 rpm. Afterward, the tissue was washed repeatedly in PBS. Specimens were then re-mounted, viewed, and photographed using standard exposures on a IX71 epi-fluorescence microscope (Olympus, Tokyo, Japan) to check that the immunoreactivity of the previous layer had been effectively eluted. Next, tissue was cleared with 0.5% Triton X-100 in PBS overnight before the next layer of primary and secondary antisera was applied (Table 1).

**TABLE 1:** Primary and secondary antibody details.

	Species	Manufacturer	Ref. number	Concentration used	Duration
**First layer, primary antibodies**					
NF200	Mouse	Sigma	N0142	1:1,000	3 days
MBP	Rat	Merk	MAB 395	1:200	3 days
vWF	Rabbit	Chemicon	AB7356	1:100	3 days
**First layer, secondary antibodies**					
CY3	Donkey-anti mouse	Jackson	715-165-151	1:400	Overnight
CY5	Donkey-anti rat	Jackson	55607	1:200	Overnight
AF488	Donkey-anti rabbit	Life Technologies	A21206	1:1,000	Overnight
**Second layer, primary antibodies**					
NF200	Mouse	Sigma	N0142	1:1,000	3 days
MBP	Rat	Merk	MAB 395	1:200	3 days
GLUT1	Rabbit	AbCam	Ab115730	1:500	3 days
**Second layer, secondary antibodies**					
AF647	Donkey-anti mouse			1:1,000	Overnight
AF555	Donkey-anti rabbit			1:1,000	Overnight
AF488	Donkey-anti-rat			1:1,000	Overnight

NF200, Neurofilament-H; MBP, myelin basic protein; vWF, von Willebrand factor; GLUT1, glucose transporter 1.

### Sudan Black B staining

Some specimens were stained with Sudan Black B (Sigma, St. Louis, MO, United States) to reveal myelinated axons. Specimens were passed through increasing concentrations of ethanol (30%→50%→70%) with 1 min incubations at each concentration, followed by 15 min in Sudan Black B (dissolved in 70% ethanol). The specimen was then washed in PBS for 10 min before immersion in buffered glycerol diluted with PBS (50%→70%→100%, pH 8.6) and mounting on glass slides.

### Microscopy image and statistical analysis

Immunohistochemically labelled preparations were scanned using an Olympus VS200 slide scanner (Olympus, Tokyo, Japan) allowing low power, high resolution magnification of large specimens. All preparations were stained with neurofilament-H (NF200), myelin basic protein (MBP; or Sudan Black B), and glucose transporter 1 (GLUT1). Analysis of the width of ascending nerves, internodal strands and colonic nerves were measured using QuPath software (University of Edinburgh, version 0.3.0; Bankhead et al., 2017). Three patient samples (one descending colon, one sigmoid colon, and one rectum) were stained for von Willebrand factor (VWF) in combination with NF200 and MBP. Images were also captured at 20× (NA: 0.8) and 40× (NA: 1.1) magnification using a LSM880 confocal microscope (Zeiss, Oberkochen, Deutschland) and analysed using ImageJ software (version 2.1.0, Bethesda, MD, United States). An unpaired Mann–Whitney *U* test was used to compare NF200 widths of internodal strands and ascending nerves. A Kruskal–Wallis test, adjusted with Dunn’s test for multiple comparisons, was used to compare widths of ascending nerves between regions, numbers of myelinated axons by region, and counts of colonic nerves entering specimens. An unpaired *t*-test was used to compare GLUT1 widths of colonic nerves and ascending nerves. All statistical analyses were performed in Graphpad Prism (version 9, San Diego, CA, United States). Data are presented as mean ± standard deviation unless otherwise stated.

## Results

### Histological description of ascending nerves

**FIGURE 1:**
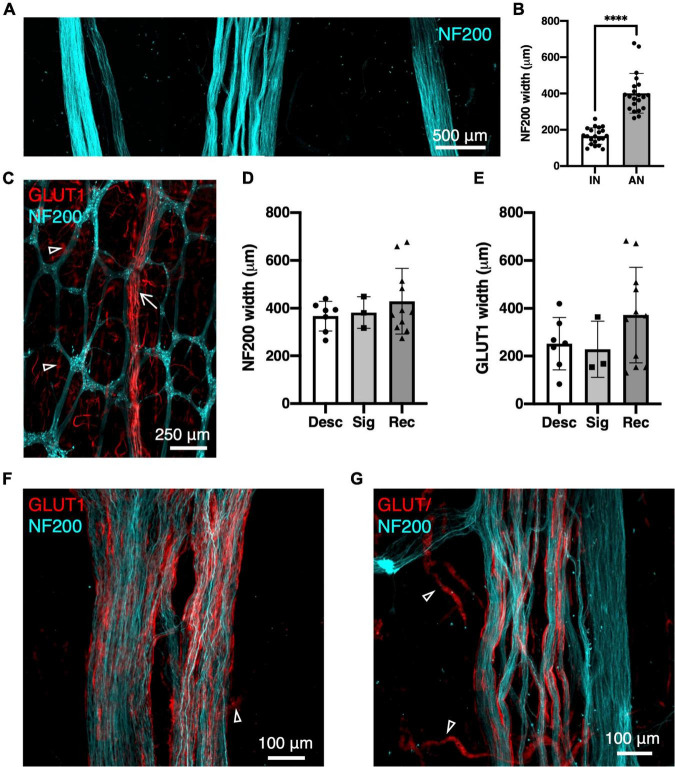
Ascending nerves labelled with neurofilament-H (NF200; cyan) and glucose transporter 1 (GLUT1; red) in human colon. **(A)** An ascending nerve (middle) is wider and has several rope-like strands, compared to two internodal strands of the myenteric plexus (left and right). **(B)** Summarised data for widths of 21 internodal strands (IN, *n* = 7 patients) and 21 ascending nerves (AN, *n* = 7), ****p < 0.0001. **(C)** Low power image of myenteric plexus with enteric neurons labelled with NF200 and an ascending nerve (arrow) running longitudinally, mostly independently of ganglia. **(D,E)** Widths of ascending nerves did not differ significantly between descending colon (Desc), sigmoid colon (Sig), and rectosigmoid/rectum (Rec) when measured from NF200 labelling of axons **(D)** or GLUT1 labelling of perineurial sheaths **(E)**. Panel **(F)** shows an ascending nerve in rectum which has GLUT1 immunoreactive perineurial staining across its full width. **(G)** More proximally, in descending colon the perineurial sheath is restricted to the left; on the right is an enteric internodal strand running parallel, which lacks GLUT1. Fragmented GLUT1 (open arrowheads) is labelling of red blood cells in small vessels.

Neurofilament-H was used as a general marker for the myenteric plexus as it intensely labels a small majority of nerve cell bodies and axons in human colonic myenteric plexus. Wholemount preparations displayed the typical stellate appearance of regularly spaced ganglia (Figure 1C), interconnected by internodal strands (Christensen et al., 1984). Ascending nerves were often distinguishable from regular internodal strands because they rarely entered ganglia directly, and instead followed a very straight course longitudinally throughout the length of the specimen. Ascending nerves often divided into multiple parallel strands, giving them a rope-like appearance, with bundles of strands woven loosely together (Figure 1A). The width of ascending nerves, defined by NF200 staining, was 401 ± 110 μm (21 ascending nerves from *n* = 7 patients), which was more than double the average width of internodal strands (166 ± 45 μm; 21 internodal strands from *n* = 7 patients, Figure 1B, *p* < 0.0001, Mann–Whitney *U* test, *U* = 0). When the widths of ascending nerves were compared between regions of the colon, no differences were observed between descending colon, sigmoid colon, and rectum, when measuring either NF200 (Figure 1D) or GLUT1 (Figure 1E) width (Table 2).

**TABLE 2:** The width of ascending nerves by neurofilament-H (NF200) and glucose transporter 1 (GLUT1) and number of myelinated fibres within ascending nerves separated by region of large bowel.

	Number of patient specimens	Number of ascending nerves	Mean NF200 width (range)	Mean GLUT1 width (range)	Mean # of myelinated fibres (range)
Descending colon	3	6	366.2 (246.4–439.4)	251.5 (82.9–419.7)	2.8 (0–7)
Sigmoid colon	2	3	381.7 (316.0–449.0)	228.2 (153.6–363.5)	1.3 (0–3)
Rectosigmoid/Rectum	2	11	429.1 (273.7–676.8)	371.8 (130.6–682.1)	1.9 (0–16)

Proximal colon specimens (*n* = 3 ascending colon/start of transverse colon) are excluded as they contained no obvious ascending nerves.

#### Myelinated axons

**FIGURE 2:**
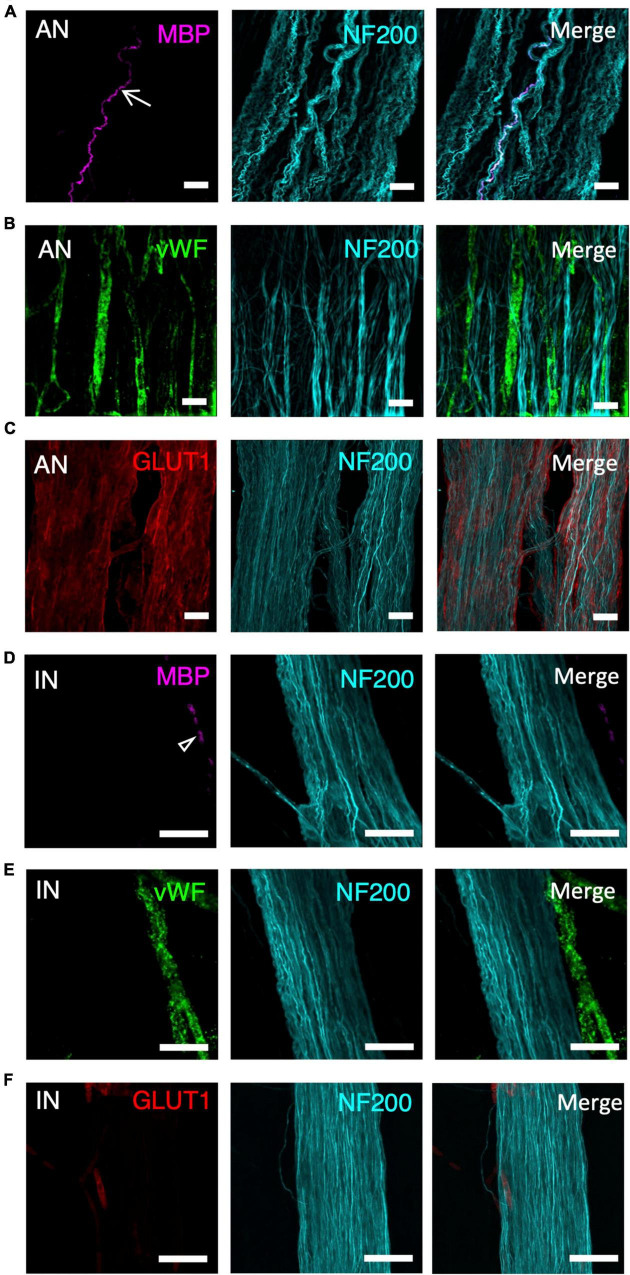
A panel of markers, applied with neurofilament-H (NF200), was used to differentiate ascending nerves (AN) from internodal strands (IN) shown at high magnification (40×). This panel included myelin basic protein (MBP- A-AN, D-IN), von Willebrand factor (vWF- B-AN, E-IN), glucose transporter 1 (GLUT1- C-AN, F-IN), and neurofilament-H (NF200). **(A,D)** Arrow indicates a myelinated axon—these were not present in all ascending nerves **(A)**. No myelinated axons are labelled in the internodal strand **(D)** although there is some fluorescence from dense collections of red blood cells (open arrowhead). **(B,E)** vWF fills several blood vessels running between multiple strands of an ascending nerve **(B)**. In contrast, vWF labelling lies outside the internodal strand **(E)**. **(C,F)** GLUT1 labels a multi-stranded ascending nerve that fills most of the image **(C)**, whereas internodal strands rarely showed GLUT1 immunoreactivity; when present it was confined to small, isolated patches **(F)**. All scale bars = 50 μM.

Ascending nerves often contained a subset of myelinated axons, which could be labelled by either Sudan Black B (Supplementary Figure 1) or MBP immunohistochemistry (Figure 2A). The number of myelinated axons ranged from 1 to 16 in a single ascending nerve. Not every ascending nerve contained myelinated axons; 8 out of 20 identified ascending nerves had no myelinated axons within the length of the specimen. This suggests that myelin is not a reliable marker for ascending nerves. When present, myelin typically occurred as lines of stained Schwann cells separated by small unlabelled nodes of Ranvier. However, in some axons, myelination was irregular or discontinuous (Supplementary Figure 2). There were no significant differences in the number of myelinated axons in ascending nerves in different regions of the colon (Table 2). Myelinated axons were not detected in regular internodal strands or ganglia of the myenteric plexus, except when they arose from ascending nerves (Figure 2D).

#### Blood vessels

Ascending nerves contain more vascular elements than regular internodal strands. Staining with vWF showed many small blood vessels running along with—and looping through—the bundles of axons that make up the ascending nerves (Figure 2B). In comparison, blood vessels did not run within regular internodal strands and, when present, ran down the outside of the strand, often in a different plane to the bundle of axons (Figure 2E). In wholemount preparations it was often necessary to generate a *z*-stack image with confocal microscopy to demonstrate that blood vessels ran within the ascending nerve rather than on the surface.

#### Perineurial sheath

The glucose transporter, GLUT1, is highly expressed by perineurial cells of extrinsic nerves but minimally expressed in the intrinsic nervous system of the gut (Kakita et al., 2000). GLUT1 labelling revealed a hollow, tube-like sheath encasing the bundles of axons that formed ascending nerves within the wall of the colon (Figure 2C) which had been tentatively identified based on NF200 staining (width, straightness, and multi-stranded appearance). The GLUT1 staining spanned the entire width of ascending nerves identified in the rectosigmoid and rectal specimens (Figure 1F). However, more proximally, in descending colon and upper sigmoid colon, GLUT1 only labelled some bundles within the ascending nerve (Figure 1G). This suggests that as the ascending nerves travel proximally, regular enteric internodal strands join the ascending nerves and run with them. Regular internodal strands rarely showed any GLUT1 positive staining; the occasional exceptions were very short sections between ganglia (Figure 2F). GLUT1 was also strongly expressed in the cell membranes of red blood cells, providing a positive control for staining in each preparation (Supplementary Figure 3).

### Location and number of ascending nerves

Eleven specimens consisting of the full circumference of the colon were dissected and labelled for NF200, MBP, and GLUT1 immunoreactivity. One or more ascending nerves was identified in 7 of the 11 specimens (regions of the 7 specimens include 3 descending, 2 sigmoid, 1 rectosigmoid, and 1 rectum). The three specimens from the right side of the colon (2 ascending and 1 ascending/start of transverse) had no identifiable ascending nerves (images of specimens H2363, H2370, and H2407 will be available by Pennsieve DOI: 10.26275/vs5w_fua4). One specimen of sigmoid colon was notably difficult to dissect, leading to poor antibody penetration and patchy labelling. This preparation did not have an identifiable ascending nerve at any point around the circumference.

**FIGURE 3:**
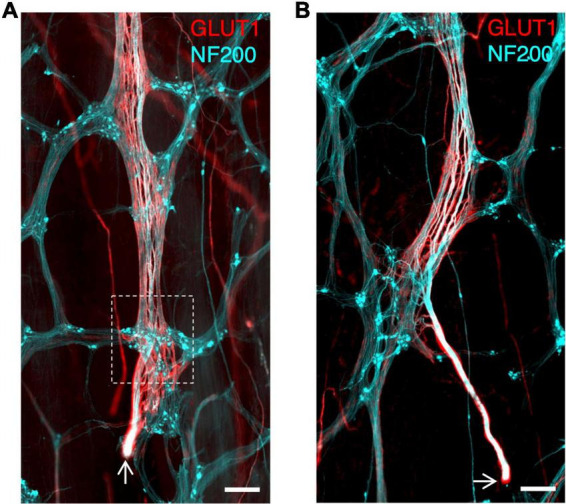
**(A,B)** Ascending nerves enter the bowel wall in the rectosigmoid region. Arrows indicate the cut end of the nerve where it was severed during surgery; this cut end lies outside the bowel wall. The myenteric plexus is stained with neurofilament-H (NF200; cyan) as are axons in the ascending nerve. The perineurial sheath of the ascending nerve is labelled with glucose transporter 1 (GLUT1; red). The ascending nerve runs underneath the first myenteric ganglion it encounters (See Supplementary Figure 4 for an animation to visualise the plane of the ascending nerve beneath the ganglion) (white dashed box). All scale bars = 500 μm.

In the 7 specimens with identified ascending nerves, the number ranged between 1 and 11 around the circumference. The descending colon and sigmoid colon had 1–3 ascending nerves, with one often located near the mesenteric taenia coli. One of our descending colon specimens was collected from just below the splenic flexure. In this specimen’s full circumference two obvious ascending nerves were identified, suggesting ascending nerves travel proximally at least as far as the proximal parts of the descending colon. One rectosigmoid and one rectum specimen had 11 and 6 ascending nerves, respectively, which were spread around the specimens’ circumference. In the rectosigmoid specimen 4 of the 11 ascending nerves were labelled back to their severed end where they entered the bowel wall, confirming that these nerves are of extrinsic origin (Figures 3A,B and Supplementary Figure 4).

### Comparison to colonic nerves

**FIGURE 4:**
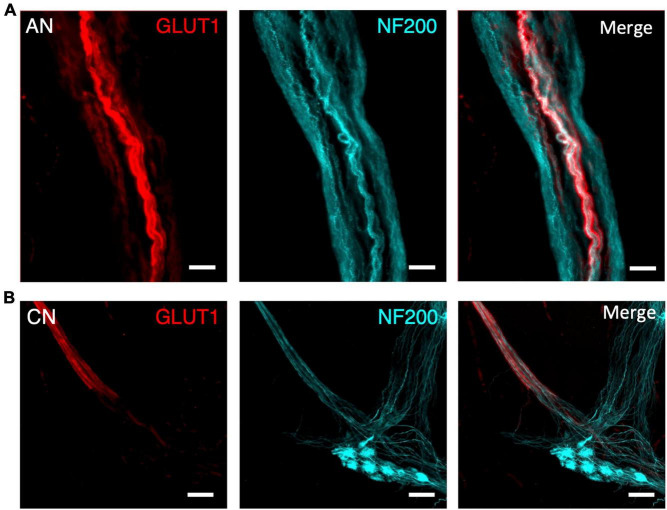
Comparison of an ascending nerve (AN) **(A)** and colonic nerve (CN) **(B)** in the bowel wall of the same sigmoid colon specimen, labelled for glucose transporter 1 (GLUT1; red) and neurofilament-H (NF200, cyan). Both nerve bundles are surrounded with a GLUT1-immunoreactive sheath, however, the ascending nerve runs the full length of the image. In comparison, the colonic nerve loses its GLUT1 labelling when its axons enter a myenteric ganglion. All scale bars = 100 μm.

Lumbar colonic nerves are sympathetic nerves which arise from the inferior mesenteric ganglia and project to the gut wall, often via paravascular nerve trunks. These were also GLUT1 immunoreactive until just beyond the point where they entered the wall of the bowel, but were readily distinguishable from ascending nerves (Figure 4A). Typically, they were much narrower than ascending nerves [60 ± 25 μm from 10 colonic nerves (*n* = 10 patients) compared to 311 ± 171 from 21 ascending nerves (*n* = 7 patients), *p* < 0.0001, unpaired *t*-test, *T* = 4.6], their axons run circumferentially and obliquely and only short distances longitudinally, and they lost GLUT1 immunoreactivity within a short distance (1 mm) of their axons joining the myenteric plexus (Figure 4B). The number of colonic nerves entering different regions of human large intestine was analysed, based on GLUT1 immunoreactivity. The density of colonic nerves in colon appeared to vary with ascending/transverse colon: (0.14 ± 0.16 nerves per cm^2^, *n* = 3), descending colon: (0.72 ± 0.25, *n* = 3), sigmoid colon: (0.73 ± 0.25, *n* = 3), and rectosigmoid/rectum: (0.47 ± 0.45, *n* = 2). More lumbar colonic nerves entered close to the middle (mesenteric) taenia (0.85 ± 0.58 nerves per cm^2^) rather than the left (0.26 ± 0.35 nerves per cm^2^; *p* = 0.03, Kruskal–Wallis test, *H* = 7.8) or right taenia (0.28 ± 0.32 nerves per cm^2^, *p* = 0.07, Kruskal–Wallis test, *H* = 7.8) (assessment based on 10 specimens of full circumference separated into 3 similar sized segments).

### Rapid anterograde tracing

**FIGURE 5:**
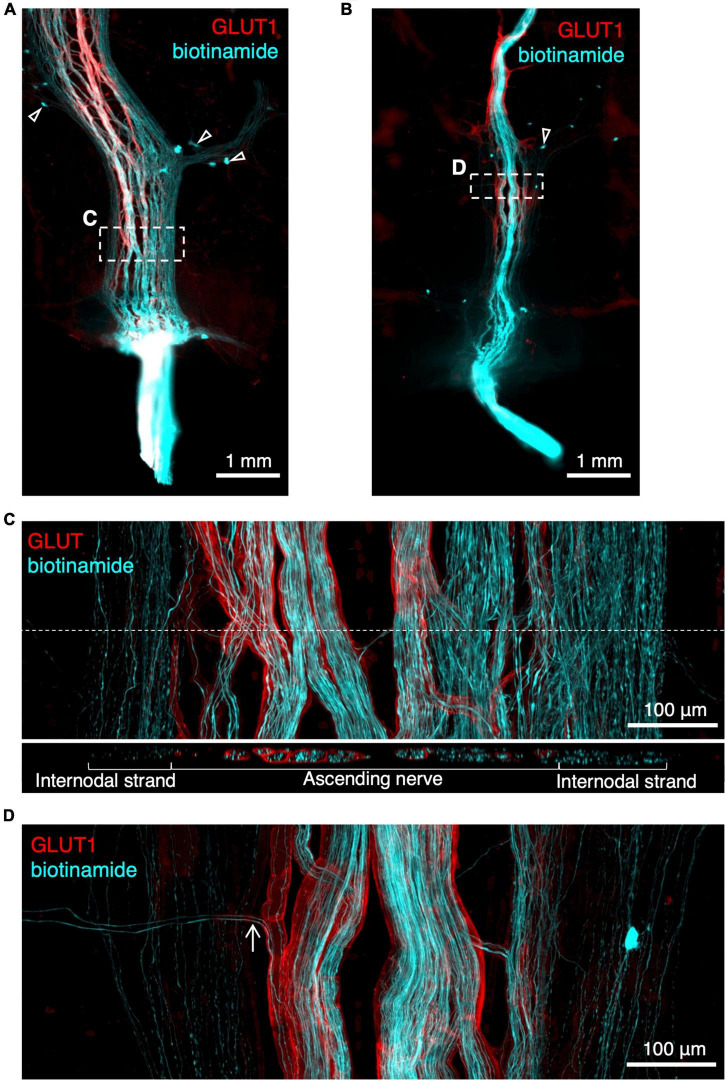
Rapid anterograde tracing of ascending nerves with biotinamide (cyan) combined with glucose transporter 1 (GLUT1) immunoreactivity (red). An ascending nerve was dissected free for ∼5 mm at the aboral end of a segment of colon and labelled with biotinamide (see section “Materials and methods”). As expected, axons running within a GLUT1 immunoreactive sheath were labelled. Panel **(A,B)** show biotinamide fills at low power. Note numerous small biotinamide-labelled myenteric nerve cell bodies (open arrowheads); these are likely to have been filled from nearby internodal strands of the myenteric plexus. Attempts to dissect away internodal strands prior to filling **(B)** were only partially successful. Panel **(C)** shows a detailed region from panel **(A)** (dashed outlines) with GLUT1 labelled ascending nerve and parallel internodal strands on either side. An X–Z virtual section (at the level of the dashed line) shows GLUT1 sheaths surrounding strands of the ascending nerve but not internodal strands. Panel **(D)** shows two biotinamide-filled axons (arrow) leaving an ascending nerve to join nearby myenteric plexus [dashed outline in panel **(B)**].

Biotinamide tracing has previously been used to label the course of axons in extrinsic nerves in isolated specimens of gut (Tassicker et al., 1999; Chen et al., 2015). In the present study, ascending nerves were dissected and mobilised at the distal end of specimens of human colon and labelled with biotinamide overnight. As expected, this filled numerous axons in the ascending nerve bundles over distances of up to 12 mm (Figures 5A,B). However, in each case, juxtaposed internodal strands (lacking GLUT1 immunoreactivity) close to the ascending nerves were also consistently filled. This suggests that ascending nerves may follow internodal strands in the descending and sigmoid colon (Figure 5C). Attempts to remove internodal strands and obtain selective fills of ascending nerves were only partially successful (*n* = 3; Figure 5D). For this reason, it was not possible to identify the enteric targets of parasympathetic axons, although axons leaving the ascending nerves to join the myenteric plexus were often visible (Figure 5D).

## Discussion

In this study, a panel of immunohistochemical markers was tested to identify the ascending nerves in specimens of human colon. Antisera labelled myelinated fibres (MBP), intraneural blood vessels (vWF) and the perineural sheath (GLUT1) surrounding nerve bundles, enabling their quantification in the descending colon, sigmoid colon, and rectum. No ascending nerves were detected in the ascending colon or proximal transverse colon; this is consistent with two previous human studies, which traced ascending nerves as far proximally as the middle of the transverse colon (Kumar and Phillips, 1989) or top of the descending colon (Stach, 1971). Here we report that the combination of GLUT1 and NF200 staining clearly distinguished ascending nerves from regular internodal strands of the myenteric plexus. This is due to GLUT1 being abundantly expressed in both the plasma membrane and cytoplasm of perineural cells (Gerhart and Drewes, 1990).

As reported previously, a small subset of axons within the ascending nerves are myelinated (Stach, 1971; Christensen et al., 1984; Christensen and Rick, 1987). The number of myelinated axons in our study did not differ significantly between different regions of the colon. In the cat, Christensen and Rick (1987) showed that the proportion of myelinated axons declines proximally, with 7–9% of axons in the sigmoid colon and 2–3% of axons in the transverse colon being myelinated. This discrepancy may reflect a species difference (humans vs. cats) or a difference in methodological approaches (wholemount vs. tissue sections). Myelin is a specific marker of ascending nerves in human colonic myenteric plexus as it is essentially absent from internodal strands and myenteric ganglia. However, it is not a sensitive marker as it was only present in 60% of ascending nerves, identified by GLUT1 immunoreactivity. It is likely that these myelinated axons are largely afferent in function. In recordings from ascending nerves in dog colon, it was shown that stimulation of ventral roots (primarily containing axons of efferents) activated C-fibres whereas stimulation of dorsal roots (afferents) activated both C and myelinated A-delta fibres, thus the latter are probably sensory (Fukai and Fukuda, 1984).

Studies in dogs and cats reported that ascending nerves diminish in size as they travel orally, presumably through axons leaving to connect with myenteric neurons (Stach, 1971; Fukai and Fukuda, 1984). In the present study ascending nerves were seen to branch occasionally and axons appeared to leave the trunks and enter the myenteric plexus, however, no significant difference in width of ascending nerves (measured from NF200 immunoreactivity) was seen. Christensen and Rick (1987) reported in cats that the total number of axons was greatest approximately 5 cm proximal to the rectum without measurable decreases as the nerve passed further orally. Here, we showed that GLUT1-immunoreactive ascending nerves were usually associated with enteric internodal strands which may exaggerate their apparent width. To investigate this further, we measured the width of GLUT1 staining without reference to NF200; there was a slight trend for wider ascending nerves in the rectum compared to the descending colon and sigmoid colon, however, this did not reach statistical significance, perhaps because of the large variation of widths within each region and/or small sample sizes.

Ascending nerves arise from branches of the pelvic plexus that enter the bowel near the rectosigmoid junction, and then continue either aborally toward the anal canal or orally toward the transverse colon (Christensen, 1990). Four such entry points, with severed ends of nerve trunks, were identified in the present study (see Figure 3). Consistent with this, we found that there were more ascending nerves in the rectosigmoid preparation (11 ascending nerves) and rectal specimen (6 ascending nerves) than in more proximal regions of the colon. The number of ascending nerves around the circumference in human colon has previously been estimated as 2–3 (Kumar and Phillips, 1989).

The effect of activation of sympathetic and parasympathetic pathways in the function of the colorectum is reasonably well established. Langley and Anderson (1895) showed in cats and dogs that stimulation of sympathetic pathways *via* lumbar ventral nerve roots caused inhibition of distal colon and contraction of the internal anal sphincter, whereas parasympathetic pathways stimulated at sacral roots caused contractile activity of both colon and rectum. In cats, imaging studies showed that chronic lesions to sympathetic pathways caused a small increase in colonic contractility whereas lesioning sacral parasympathetic pathways disrupted defecatory activity and led to distal obstruction (M’Fadden et al., 1935). Acutely, lesioning sympathetic pathways in cats had little effect on motility but severing sacral nerves led to reduced activity and constipation (Garry, 1933a). From studies of human patients with pelvic lesions, it was concluded that sacral spinal reflex pathways augment enteric reflex pathways activated by rectal distension, leading to powerful rectal contractions and anal relaxations. This was not the case for sympathetic pathways (Denny-Brown and Robertson, 1935). In cats, rectal distension activates defecatory pathways, causing contraction of the upstream colon and this reflex is largely dependent on intact pelvic pathways (Garry, 1933b). Similarly in rabbits, activation of pelvic parasympathetic pathways evokes colonic contractions, whereas contractility is inhibited by stimulation of the sympathetic lumbar outflow. Stimulation of rabbit distal bowel activates colonic motility orally and again, this is largely (but not entirely) dependent on intact extrinsic pelvic pathways (Garry and Gillespie, 1955). These studies suggest that sacral parasympathetic pathways, acting *via* ascending nerves, have excitatory effects on large bowel motility together with contributing to anal sphincter relaxation.

Focussing on the ascending nerves, evidence for their functional significance was provided by recording bursts of action potentials in axons in the ascending nerves which were temporally associated with spontaneous contractions of the colon (De Groat and Krier, 1978). In an elegant study, Fukai and Fukuda (1984) showed that the spread of excitation orally in the colon from a stimulus is mediated by intramural pathways; most likely the ascending nerves. Responses in upper colon were abolished by tightening a ligature around the gut more anally—a lesion that kept extrinsic pathways intact but which interrupted intramural pathways.

Taken together these studies show that extrinsic pelvic nerves contain both afferent and efferent nerve fibres which mediate powerful extrinsic reflexes, which augment polarised enteric reflex pathways (Bayliss and Starling, 1900) over considerable lengths of the distal colon. Our study has characterised the anatomical substrates for these parasympathetic pathways in human colon as the ascending nerves labelled with GLUT1. We have also shown that sympathetic pathways arising from lumbar colonic nerves are also GLUT1-immunoreactive for short distances within the wall of the colon.

The present study identified the entry points for some ascending nerves in the rectosigmoid region, by the presence of severed endings on the surface of the excised tissue. This extrinsic origin is consistent with presence of GLUT1 immunoreactivity, which is a marker of perineurium in extrinsic nerve trunks (Muona et al., 1993) but not the myenteric plexus. Severing these nerves outside the bowel wall is inevitable during low anterior resections to remove rectal carcinoma. Following low rectal excision, severe symptoms develop in a majority of patients; these include incontinence, altered stool frequency, urgency, dysfunction of defecation, poor gas–stool discrimination, and clustering of defecation. This collection of symptoms is known as “low anterior resection syndrome” or LARS (Keane et al., 2017). While it has been suggested that loss of rectal reservoir function may contribute to LARS, it is also possible that colonic resection may alter distal colonic motility. The rectosigmoid junction is the primary site of origin for the cyclic motor pattern (Dinning et al., 2014), which has been speculated to act as a rectosigmoid brake (Lin et al., 2017). An absent or diminished cyclic motor patterns has been shown in patients with faecal incontinence (Patton et al., 2013). In patients with LARS a similar distal colonic reduction in the cyclic motor pattern has been shown along with altered colonic motility in other regions (Keane et al., 2021; Asnong et al., 2022). Therefore, it is possible that disruption of parasympathetic input to enteric neuronal pathways in the distal colon may be a factor in these changes.

We speculate that in patients with LARS, the low anterior resection may sever rectal nerves before they enter the bowel wall and thus hinder re-growth and re-connection of their axons. If the site of anastomosis is above the entry point, it may be possible for axons in ascending nerves to regrow across the lesion and thus recover some functional continuity, in the months after the operation. Normal colonic motility, with propagating contractions across the site of the anastomosis, has been shown in patients who have undergone an anterior resections and regained normal bowel function (Vather et al., 2016). In addition, a recent meta-analysis identified low tumour height (measured relative to the anal verge) as a positive risk factor for major LARS (Croese et al., 2018).

The likely targets of the efferent axons of the ascending nerves are myenteric neurons located further orally in the colon. Whether specific functional classes of enteric neurons are targetted is not clear. In the present study, it was not possible to examine this possibility because biotinamide fills of ascending nerves were consistently contaminated by fills of enteric axons in juxtaposed internodal strands. Identifying the pathways activated by parasympathetic axons in ascending nerves is a significant challenge for future studies.

To summarise, we show that using a panel of markers to identify ascending nerves in the human colon was a more reliable approach than using a single marker. The combination of NF200 and GLUT1 was found to be as effective as using all four markers. Data on the location and extent of ascending nerves may be of assistance in future studies, including the possibility of studying effects of stimulating them within the bowel wall.

## Data availability statement

The original contributions presented in the study are publicly available through the Pennsieve platform after curation by the Pennsieve staff at the following link: DOI: 10.26275/vs5w_fua. Further inquiries can be directed to the corresponding author.

## Ethics statement

The studies involving human participants were reviewed and approved by the Southern Adelaide Clinical Human Research Ethics approval number 207.17. The patients/participants provided their written informed consent to participate in this study.

## Author contributions

MJ and AH conducted the research as joint leads and wrote the manuscript. MC, DW, TS, PD, and SB developed the study concept and design. RP contributed to the laboratory experiments. All authors reviewed the final manuscript and images, contributed to the article, and approved the submitted version.
