# Supplementary material for: Characterisation of parasympathetic ascending nerves in human colon
Source: Front Neurosci. 2022 Dec 1;16:1072002. doi: 10.3389/fnins.2022.1072002 (PMC9752816; doi:10.3389/fnins.2022.1072002)
Supplement: Supplementary file 1 [file Data_Sheet_1.zip › Data Sheet 1.PDF]

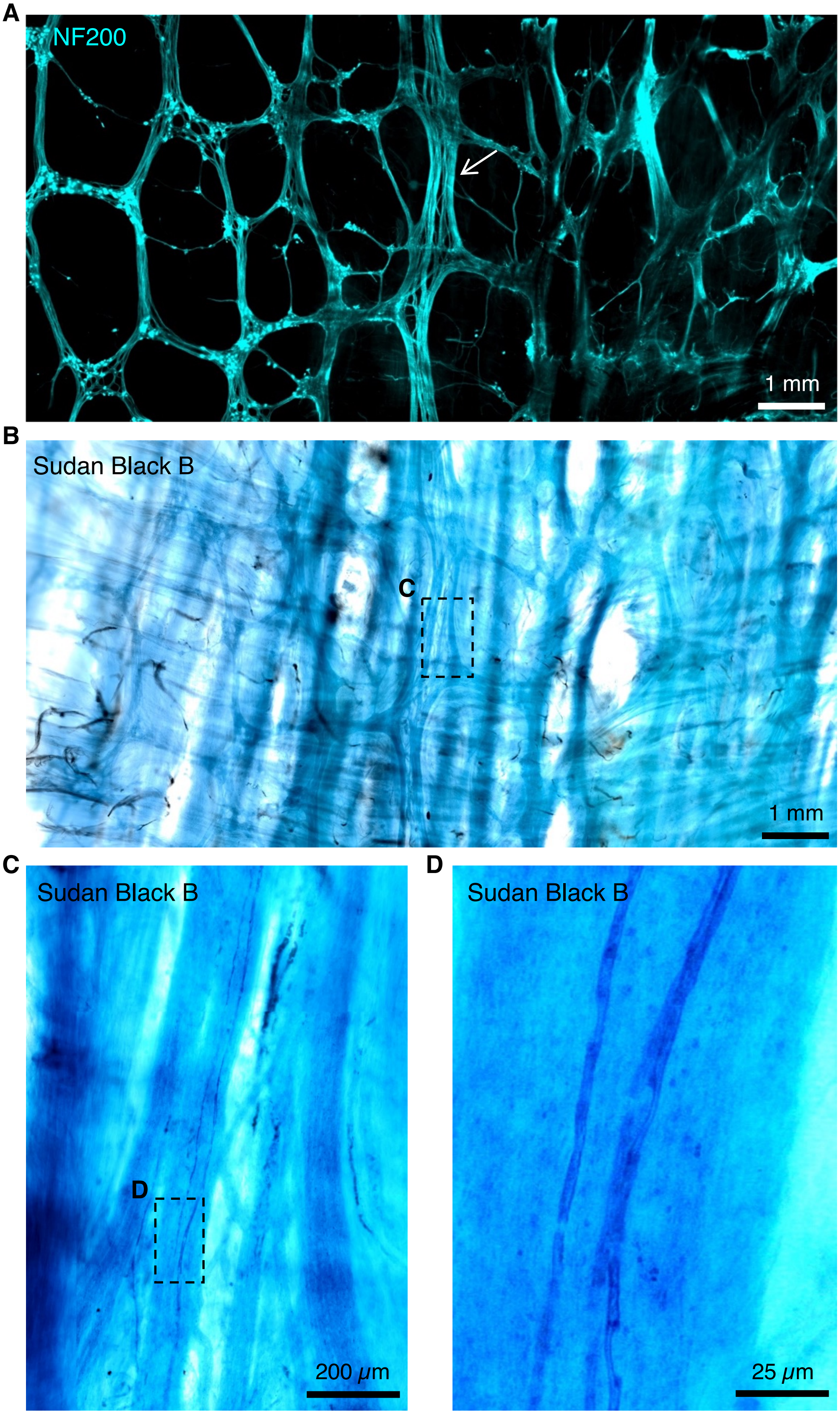

Supplementary Figure 1. (A) neurofilament-H (NF200; cyan) staining of myenteric plexus revealed an ascending nerve in descending colon (arrow). **(B)** Sudan Black B staining outlined the same structure. **(C&D)** higher magnification images of the ascending nerve showed Sudan Black B stained myelinated axons running within the ascending nerve.
