# Supplementary material for: Characterisation of parasympathetic ascending nerves in human colon
Source: Front Neurosci. 2022 Dec 1;16:1072002. doi: 10.3389/fnins.2022.1072002 (PMC9752816; doi:10.3389/fnins.2022.1072002)
Supplement: Supplementary file 1 [file Data_Sheet_1.zip › Data Sheet 2.PDF]

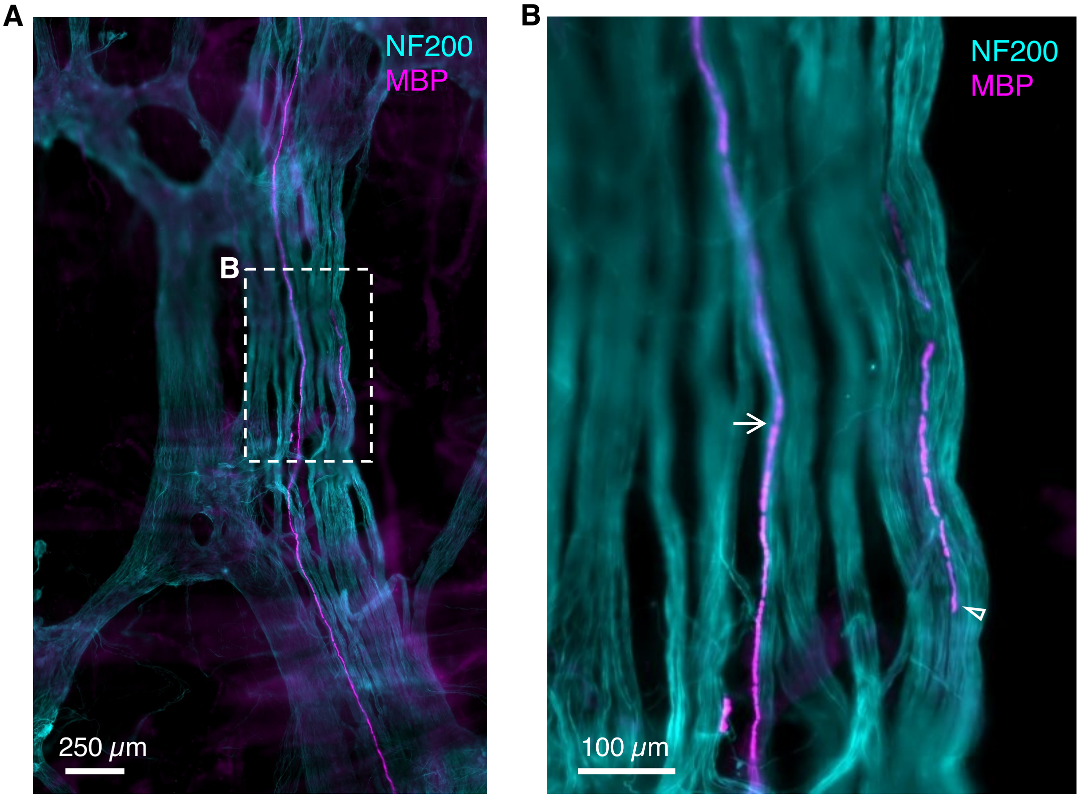

Supplementary Figure 2. (A) an ascending nerve stained with neurofilament-H (NF200; cyan) and myelin basic protein (MBP; magenta). **(B)** In the higher magnification image (dashed outline in A) the arrow points to a continuous myelinated axon and the unfilled arrowhead indicates a discontinuous myelinated axon.
