# Supplementary material for: Characterisation of parasympathetic ascending nerves in human colon
Source: Front Neurosci. 2022 Dec 1;16:1072002. doi: 10.3389/fnins.2022.1072002 (PMC9752816; doi:10.3389/fnins.2022.1072002)
Supplement: Supplementary file 1 [file Data_Sheet_1.zip › Data Sheet 3.PDF]

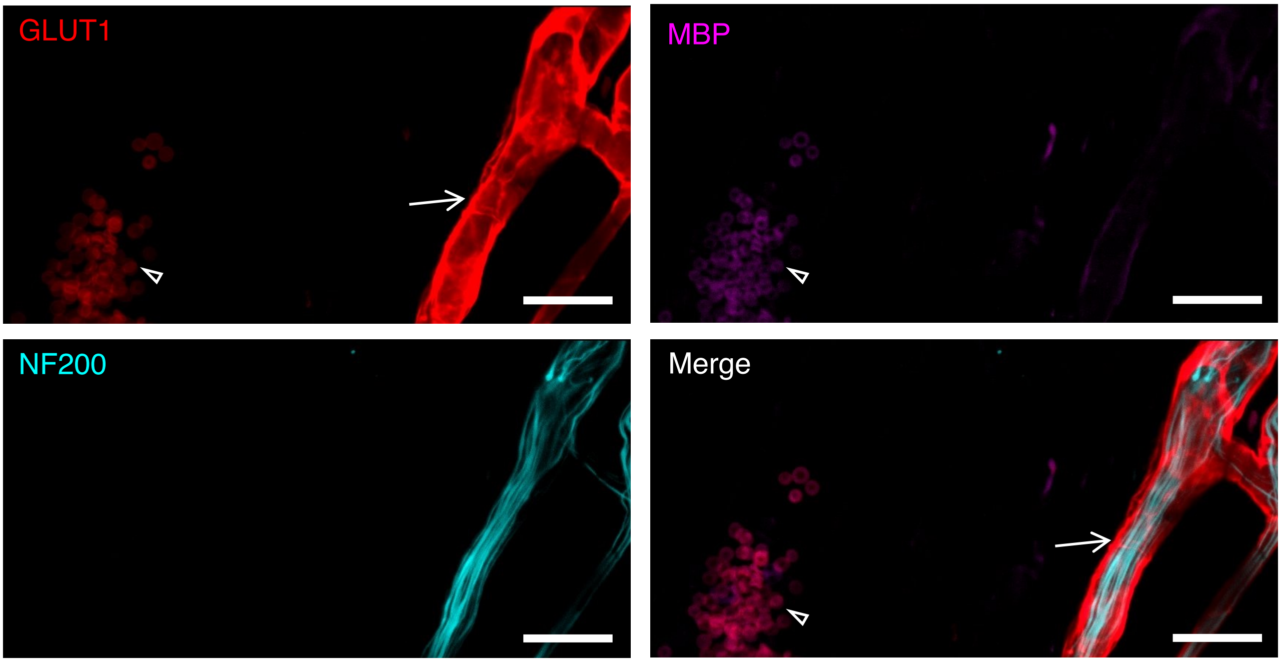


**Supplementary Figure 3.** An ascending nerve stained with glucose transporter 1 (GLUT1; red), myelin basic protein (MBP; magenta) and neurofilament-H (NF200; cyan). The pattern of GLUT1 staining differed when it labelled perineural cells forming the perineural sheath surrounding ascending nerves (arrow) compared to the cell membrane of blood cells (arrowhead). The same blood cells also stained with MBP (arrowhead). All scale bars = 50 µM.
